# Coordinated outreach for veterans in long-term care facilities by an integrated Veterans Affairs healthcare system during the COVID-19 pandemic

**DOI:** 10.1017/ice.2020.326

**Published:** 2020-07-03

**Authors:** Alexander Winnett, Lauren P. Jatt, Linda Sohn, Marcia Lysaght, Thomas Yoshikawa, Steven R. Simon, Christopher J. Graber, Matthew Bidwell Goetz

**Affiliations:** 1 David Geffen School of Medicine, University of California Los Angeles, Los Angeles, California; 2 Veterans Affairs Greater Los Angeles Healthcare System, Los Angeles, California

*To the Editor*—We read with interest the article by Guar et al^1^, highlighting the burdensome and dangerous effect of the coronavirus disease 2019 (COVID-19) pandemic on long-term care facilities (LTCFs), and their recommendations to support these vulnerable populations. Even large healthcare organizations have struggled to obtain the resources necessary to maintain normal operations,^2^ and these challenges may be accentuated in community LTCFs with fewer staff and less financial flexibility to obtain resources for the prevention and control of severe acute respiratory coronavirus virus 2 (SARS-CoV-2) outbreaks. In response to the sentiment described by Guar et al^1^ that “these extraordinary times call for unprecedented measures to protect our vulnerable LTCF residents” and their recommendation for “hospital systems to include LTCF settings as high-priority sites for increased access to respiratory viral tests, including for SARS-Cov-2, and [personal protective equipment],” we offer a brief description of unprecedented measures by a large, integrated, Veterans Affairs (VA) healthcare system to support community LTCFs facing resource limitations during the COVID-19 pandemic.

The VA Greater Los Angeles Healthcare System (VAGLAHS) is a multicampus healthcare system that includes a tertiary-care hospital, several satellite clinics, and on-campus LTCFs. In response to the COVID-19 outbreak amongst residents of 2 of the on-campus LTCF wards, 1 of these LTCF wards was converted to a COVID-19 recovery unit (CRU) to provide subacute medical care specifically for SARS-CoV-2–positive individuals following acute-care hospitalization, separate from uninfected LTCF residents.^3^ During this outbreak, VAGLAHS had access to high-volume molecular diagnostic testing capacity and utilized universal, serial, surveillance testing of residents to identify and isolate individuals at risk of transmitting the virus. These resources allowed for effective control practices ultimately halting the outbreak, with no new infections after only 2 weeks.^4^

However, many veterans reside in community LTCFs that are also at risk of a COVID-19 outbreak but may have more limited access to resources needed to prevent or control an outbreak, such as high-volume testing and personal protective equipment. Recognizing this dilemma, VAGLAHS leadership followed the provisions of the “Fourth Mission” of the VA to help community facilities also dealing with the pandemic: to “improve the Nation’s preparedness for response to war, terrorism, national emergencies, and natural disasters by developing plans and taking actions to ensure continued service to veterans, as well as to support national, state, and local emergency management, public health, safety and homeland security efforts.”^5^ A VAGLAHS Long-Term Care COVID-19 (LTCCV19) working group was established to take action and coordinate outreach efforts.

The LTCCV19 surveyed the leadership of 25 community LTCFs to assess needs and identify areas of potential assistance; 18 facilities responded, and to date, the VAGLAHS LTCCV19 has engaged in targeted support with 11 facilities. VAGLAHS staff completed twice daily telephone calls with the community facilities’ administrators for real-time feedback on new cases of COVID-19 in residents and staff. For 7 facilities with testing limitations, the VAGLAHS LTCCV19 deployed registered nurses to assist in collecting specimens from veteran residents and to perform universal SARS-CoV-2 surveillance testing, and train community staff on specimen collection. Veterans with COVID-19 from community LTCFs have been admitted to the acute-care VA hospital for medical care, then to the CRU if their community LTCFs were not able to house them upon recovery. VAGLAHS staff trained in infection control were dispatched to community LTCFs to review on-site practices and strategize areas for improvement, including those listed by Guar et al.^1^

As Guar et al^1^ emphasize, to combat this pandemic we must fortify areas of our society at higher risk of both outbreak and severe disease, such as LTCFs. The SARS-CoV-2 virus clearly does not recognize institutional boundaries, and institutions with the resources and capacity to assist in this fortification can play a substantial role in protecting vulnerable members of our community, ultimately reducing the burden of the ongoing COVID-19 pandemic.
